# High‐content screening of drug combinations of an mPGES‐1 inhibitor in multicellular tumor spheroids leads to mechanistic insights into neuroblastoma chemoresistance

**DOI:** 10.1002/1878-0261.13502

**Published:** 2023-08-21

**Authors:** Ahlem Zaghmi, Erdem Aybay, Long Jiang, Mingmei Shang, Julia Steinmetz‐Späh, Fredrik Wermeling, Per Kogner, Marina Korotkova, Päivi Östling, Per‐Johan Jakobsson, Brinton Seashore‐Ludlow, Karin Larsson

**Affiliations:** ^1^ Rheumatology Unit, Department of Medicine, Solna Karolinska Institutet, Karolinska University Hospital Stockholm Sweden; ^2^ Childhood Cancer Research Unit, Department of Women's and Children's Health Karolinska Institutet Stockholm Sweden; ^3^ Department of Oncology‐Pathology, Science for Life Laboratory Karolinska Institutet Stockholm Sweden

**Keywords:** drug screen, MCTS, mPGES‐1, neuroblastoma, PGE_2_, spheroids

## Abstract

High‐throughput drug screening enables the discovery of new anticancer drugs. Although monolayer cell cultures are commonly used for screening, their limited complexity and translational efficiency require alternative models. Three‐dimensional cell cultures, such as multicellular tumor spheroids (MCTS), mimic tumor architecture and offer promising opportunities for drug discovery. In this study, we developed a neuroblastoma MCTS model for high‐content drug screening. We also aimed to decipher the mechanisms underlying synergistic drug combinations in this disease model. Several agents from different therapeutic categories and with different mechanisms of action were tested alone or in combination with selective inhibition of prostaglandin E_2_ by pharmacological inhibition of microsomal prostaglandin E synthase‐1 (mPGES‐1). After a systematic investigation of the sensitivity of individual agents and the effects of pairwise combinations, GFP‐transfected MCTS were used in a confirmatory screen to validate the hits. Finally, inhibitory effects on multidrug resistance proteins were examined. In summary, we demonstrate how MCTS‐based high‐throughput drug screening has the potential to uncover effective drug combinations and provide insights into the mechanism of synergy between an mPGES‐1 inhibitor and chemotherapeutic agents.

## Graphical abstract

This study highlights the value of spheroids for screening drug combinations and selecting appropriate hits for preclinical testing. A low‐cost spheroid model was developed that combines viability testing and confocal imaging to increase the reliability of results. Further research is needed to fully investigate the mechanisms underlying the synergies between microsomal prostaglandin E synthase‐1 inhibitors and chemotherapeutic agents.

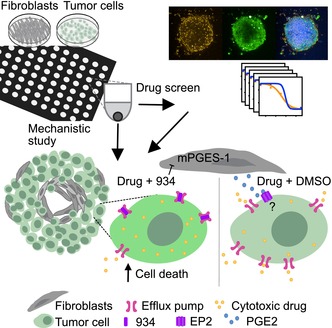

**Abbreviations**
ABC: ATP‐binding cassette
ABCB1: ABC subfamily B member 1 (also MDR1)
ABCC1: ABC subfamily C member 1 (also MRP1)
BCRP: breast cancer resistance protein
BztCl: benzethonium chloride
CAFs: cancer‐associated fibroblasts
CETSA: cellular thermal shift assay
COX: cyclooxygenase
DAB: 3, 3‐diaminobenzidine
DSS: drug sensitivity score
EthD‐1: ethidium homodimer‐1
FAP: fibroblast activation protein
FSP‐1: fibroblast‐specific protein 1
MCTS: multicellular tumor spheroids
MDR1: multidrug resistance protein 1 (ABCB1 and P‐Gp)
mPGES‐1: microsomal prostaglandin E synthase‐1
MRP1: multidrug resistance‐associated protein 1 (ABCC1)
NB: neuroblastoma
NHDF: normal human dermal fibroblasts
PDGFRb: platelet‐derived growth factor receptor beta
PGE_2_
: prostaglandin E_2_
SAP: saponin
ZIP: zero interaction potency

## 1 Introduction

The cultivation of cancer cells in the form of tumor spheroids was introduced by Sutherland et al. in the early 1970s [1]. However, their use in anticancer drug discovery has only surged in recent years due to technical advances that have enabled high‐throughput quantitative analysis of 3D cell culture models. Even though they are relatively easy to grow and treat, it is currently not trivial to collect and analyze data from image‐based assays of spheroids. This probably explains why drug screens and functional and mechanistic studies are still largely performed in 2D cultures. However, tumor cells grown in monolayers have limited ability to model critical *in vivo* features of tumors, such as cell‐to‐cell contact, nutrient availability, waste removal, pH gradients, oxygen tension, and metabolic processes [2]. In addition, cancer cells cultured in monolayers grow faster than cells in 3D models and in *in vivo* tumors, which sensitizes them to drugs [3]. This could lead to a greater likelihood of finding agents that efficiently stop highly proliferative cells in the search for new experimental drugs [4]. While 2D drug screens are likely to identify agents that inhibit cell proliferation, screens performed in 3D models have the potential to discover additional agents that target multiple cellular mechanisms; therefore, it is important to perform drug screens in such systems.

One of the major tumor‐promoting lipid mediators involved in cancer‐associated fibroblasts (CAFs) function is prostaglandin E_2_ (PGE_2_). PGE_2_ is a bioactive lipid mediator of inflammation that is overexpressed in many tumors [5, 6]. PGE_2_ plays an important role in cancer‐promoting processes and has been associated with promoting tumor stem cell expansion [7, 8] and mediating chemoresistance [9, 10]. In neuroblastoma (NB), an endocrine tumor of early childhood, we found that CAFs are the major source of microsomal prostaglandin E synthase‐1 (mPGES‐1), the key enzyme responsible for PGE_2_ production in tumors downstream of cyclooxygenase (COX)‐1/2 [11]. Inhibition of mPGES‐1 in two NB mouse models reduced tumor growth and altered the tumor microenvironment, suggesting mPGES‐1 as a putative target in NB [12].

A major limitation of tumor spheroids is the absence of elements of the tumor microenvironment and heterotypic cell interactions. CAFs are an essential component of the tumor microenvironment and support cancer cells in multiple ways, including growth factor production, extracellular matrix deposition and remodeling, immune cell exchanges, and angiogenesis support [13]. In a previous study, we established and characterized a multicellular tumor spheroid model (MCTS) using human fibroblasts and NB cancer cells. The fibroblasts expressing several CAF markers, that is, FAP, PDGFRβ, and FSP‐1, were actively recruited into the spheroid and contributed with structure, heterotypic cell interactions, and tumor‐promoting PGE_2_ [14]. By adding a stromal component, our model partially overcame the limitations associated with tumor spheroids and enabled studies on the inhibition of mPGES‐1 [14].

In the present study, we aimed to confirm that our multicellular NB spheroid model is a useful tool for performing larger drug screens and specifically for investigating the potential synergistic effects when anticancer compounds are combined with an mPGES‐1 inhibitor. Because high‐throughput analysis of MCTS remains challenging, the strategy described is a promising paradigm for identifying effective drug combinations. The feasibility of screening against NB with MCTS was demonstrated using 116 compounds in the presence and absence of an mPGES‐1 inhibitor. Based on the sensitivity of each compound and its combination with the mPGES‐1 inhibitor 934, we selected specific effective drugs for further analysis (e.g., dose–response testing and imaging). In addition, the effect of specific drugs in combination with the mPGES‐1 inhibitor on ATP‐binding cassette (ABC) transporters gain insight into the synergistic mechanism. This study demonstrates the utility of our MCTS for the identification of effective drugs and provides new insights into fundamental processes involved in the synergy between mPGES‐1 inhibitors and conventional anticancer drugs.

## 2 Materials and methods

### 2.1 Drug library

A drug library containing 116 drugs, both experimental and approved cancer drugs, was prepared by FIMM High Throughput Biology. Sixty nanoliter of each drug dilution was spotted using an Echo550 (Beckman/Labcyte, Indianapolis, IL, USA) into low‐dead‐volume 384‐well plates (784201; Greiner, Kremsmünster, Austria). Each drug was present at five concentrations spanning a 10‐fold dilution series (e.g., 1–10 000 nm). DMSO (negative control) and benzethonium chloride (100 μm BztCl, positive control) were present in at least 10 replicates each on all plates.

### 2.2 Cell culture and MCTS formation

NB cells: SK‐N‐AS (CRL‐2137; ATCC, RRID:CVCL_1700, Manassas, VA, USA), SK‐N‐BE(2) (CRL‐2271; ATCC, RRID:CVCL_0528), SK‐N‐SH (HTB‐11; ATCC, RRID:CVCL_0531), IMR‐32 (CCL‐127; ATCC, RRID:CVCL_0346), and normal human dermal fibroblasts (NHDF, C‐12300; PromoCell, Heidelberg, Germany) were cultured in complete media (RPMI (R0883; Sigma, Saint Louis, MO, USA) supplemented with 10% heat‐inactivated FBS (F7524; Sigma), 2 mm l‐glutamine (G7513; Sigma), 100 U·mL^−1^ penicillin, and 100 μg·mL^−1^ streptomycin (15070‐063; Gibco, Billings, MT, USA), at 37 °C in a humidified 5% CO_2_ atmosphere. NHDF cells, used in the experiments, were passaged less than eight times and both cell types were regularly checked for mycoplasma infection using PCR analysis. The identities of the cell lines were verified by short tandem repeat genetic profiling using the PowerPlex 16HS kit (Promega, Madison, WI, USA) in 2019. MCTS were formed using low‐attachment plates, with one spheroid per well, without any addition of extracellular matrix proteins. To form spheroids in 384‐well plates (3830; Corning, Corning, NY, USA), 1500 SK‐N‐AS cells (1500 SK‐N‐BE(2), IMR‐32, or 2000 SK‐N‐SH cells) and 2000 NHDF cells were seeded in 50 μL complete media/well using a liquid dispenser (MultiDrop Combi; ThermoFisher, Waltham, MA, USA). Plates were centrifuged for 1 min at 100 ***g*** at RT. When using 96‐well format (4515; Corning), 2500 SK‐N‐AS cells and 5000 NHDF cells were seeded in 100 μL/well with no centrifugation.

To determine where the fibroblasts localize in the spheroids, either fibroblasts or SK‐N‐AS cells were labeled with Vybrant CM‐dil as described previously [14] and imaged in Opera Phenix (PerkinElmer, Waltham, MA, USA).

### 2.3 Drug screening and image analysis

SK‐N‐AS cells and NHDF were seeded (as described in Section 2.2) in 384‐well plates and allowed to form MCTS during 3 days of incubation. Prior to treatment, the stock solution of mPGES‐1 inhibitor (i.e., 934 [15] at 10 mm in DMSO) was diluted in complete media to a concentration of 3 μm (3× final concentration). The compound library was prepared as 1000× stocks in 384‐well plates and stored at −80 °C. Complete media containing 934 inhibitor or an equivalent volume of DMSO was added to the compound library plates to generate a 3 × final concentration using a MultiDrop (ThermoFisher). Subsequently, 25 μL of the diluted library was added to the MCTS‐containing plates using a Bravo liquid handler system (Agilent, Santa Clara, CA, USA), giving an end volume of 75 μL and a final concentration of 1 μm 934. MCTS were incubated with the compound library for 4 days and monitored in an IncuCyte® S3 system (Sartorius, Göttingen, Germany).

To visualize the spheroids, their metabolic activity, and cell death, at the end of the treatment, Calcein AM (2 μm, 14948; Cayman, Ann Arbor, MI, USA), Ethidium Homodimer‐1 (EthD‐1, 3 μm, E1169; Invitrogen, Waltham, MA, USA), and Hoechst (33 μm, B2261‐25MG; Sigma‐Aldrich, Saint Louis, MO, USA) were diluted in PBS and added to the plates (15 μL/well) and incubated for 3 h [16]. The plates were subsequently imaged in the Opera Phenix high‐content screening system (PerkinElmer). Images were analyzed in harmony software (PerkinElmer) using the Find Image Region module. Parameters describing the image region were extracted for the largest object identified in each well. After evaluation of the coefficient of variation for all wells on the plate and all DMSO wells on plate (Fig. S1), volume (μm^3^) of the image region identified from the calcein AM channel and EthD‐1 channel was used. Wells that were re‐run due to focus failure were analyzed using the same pipeline and results were merged into the plate file.

Post high‐content scanning, 60 μL of liquid was removed from each well using a liquid handler (MultiFlo FX; Agilent), and 30 μL of CellTiter‐Glo® 2D Cell Viability Assay (G9681; Promega) reagent was added using liquid handler (Multidrop; ThermoFisher). Plates were incubated at 37 °C for 20 min, followed by 10 min of rapid shaking on an orbital shaker at RT. Chemiluminescence was recorded with a multimodal plate reader (EnVision; PerkinElmer). Raw luminescence readings were merged with compound and concentration information using an in‐house R‐script and curve fitting was run using the breeze (breeze.fimm.fi) pipeline. This provides screen quality control metrics, drug–response curves, and drug sensitivity score (DSS), a modified area under the curve parameter described previously [17]. We have used the values as calculated through the portal with the following parameters: Choose Readout: inhibition, Clustering method: Euclidean, curve fitting algorithm: 4PL, and DSS method: DSS2.

### 2.4 Validation of the hits using GFP‐expressing SK‐N‐AS MCTS

#### 2.4.1 Generation of GFP+ SK‐N‐AS cells

To make GFP+ SK‐N‐AS cells, a CRISPR plasmid was used. Lentiviral particles were generated by seeding 2 × 10^6^ HEK293T cells in a 10 cm plate with 10 mL medium (DMEM supplemented with 10% FBS and 1% l‐glutamine). After approximately 24 h of culture, the medium was replaced by 5 mL of fresh medium. LentiGuide‐Puro‐P2A‐EGFP (Addgene; #137729), pMD2.G (Addgene; #12259), and psPAX2 (Addgene, Watertown, MA, USA; #12260) were mixed at 4 : 5 : 1 ratio (10 μg : 12.5 μg : 2.5 μg) and transfected using Lyovec (Invivogen, Waltham, MA, USA; #lyec) according to the manufacturer's protocol. After 12 h, the medium was replaced by 8 mL of DMEM supplemented with 30% FBS and 1% l‐glutamine. After another 36 h, the supernatant containing the virus was collected, centrifuged to remove the cell debris, and used to spin infect cells. To transduce SK‐N‐AS cells, virus supernatant (50 μL) was added to each well of a six‐well plate containing cells (1 × 10^5^) with 8 μg·mL^−1^ polybrene (Sigma‐Aldrich, Saint Louis, MO, USA; #H9268). The plate was centrifuged at 37 °C, 1200 ***g*** for 45 min. After 24 h, the virus‐containing medium was replaced with fresh medium, and the infection rate was measured by the percentage of GFP+ cells. Puromycin (Invivogen; #ant‐pr) selection (10 mg·mL^−1^) was performed for 24 h to remove the non‐infected cells. Cells were sorted on GFP expression using FACS (SONY SH800 cell sorter: SONY Biotechnology, San Jose, CA, USA) after 3 weeks to ensure good level of transduction.

#### 2.4.2 Validation of the hits

MCTS were formed, using 1125 GFP+ SK‐N‐AS cells and 1500 unlabeled NHDF cells per well in 50 μL complete media in 384‐well plates. MCTS were monitored in the IncuCyte® S3 system using bright‐field and green fluorescence image acquisition. Treatment plates were prepared and added to the MCTS on Day 3, as described in Section 2.3. Compounds that were validated included: buparlisib (1–10 000 nm), cisplatin (1–10 000 nm), paclitaxel (0.1–1000 nm), selinexor (1–10 000 nm), vinblastine (0.1–1000 nm), vinorelbine (1–10 000 nm), pevonedistat (1–10 000 nm), methotrexate (0.5–5000 nm), omacetaxine (1–10 000 nm), volasertib (0.1–1000 nm), selumetinib (1–10 000 nm), raloxifene (1–10 000 nm), rigosertib (1–10 000 nm), and topotecan (1–10 000 nm). mPGES‐1 inhibitor 934 (2 μm), verapamil (10 μm), both 934 and verapamil, or vehicle control (DMSO) was added to the treatment plates. Plates were incubated for 4 days, monitored in the IncuCyte® S3 system, and finally, spheroid growth/SK‐N‐AS viability (total integrated green fluorescence) was calculated using IncuCyte built‐in software.

### 2.5 Assessment of the synergistic effects of specific hits

Once the hits were validated, we quantitatively evaluated the drug–dose combination effects of specific compounds (paclitaxel and vincristine). Briefly, treatment plates were prepared using the Echo 550 (Beckman Coulter). In a mother plate (001‐14618; Beckman Coulter, Brea, CA, USA), each compound was prepared at eight different stock solutions (1000× concentrated solution): 934, 118, celecoxib (final concentrations ranging from 0 to 10 μm), vincristine (final concentrations ranging from 0 to 100 nm), and paclitaxel (final concentrations ranging from 0 to 500 nm); DMSO (negative control) and benzethonium chloride (100 μm BztCl, positive control) were present in at least 10 replicates each on all plates. From the mother plate, 150 nL was spotted in a low‐dead‐volume microplate (784201; Greiner, Kremsmünster, Austria). Complete culture media were added to the receiving plate (50 μL/well) using peristaltic pump (MultiDrop Combi; ThermoFisher) and then 25 μL was added to the spheroid plates containing SK‐N‐AS/NHDF co‐MCTS (3830; Corning) using an automated liquid handler (Bravo; Agilent), generating 75 μL total volume/well. Plates were incubated for 4 days, monitored in the IncuCyte® S3 system, and finally, viability was assessed using CellTiter‐Glo® 3D (G9681; Promega) reagent according to manufacturer's instructions. Results were analyzed similarly to the screen using Breeze. To assess synergy, the synergyfinder 2.0 software was used and we used zero interaction potency (ZIP) scores to estimate drug interactions [18]. To assess synergy between vincristine and indomethacin or vincristine and EP antagonists (ONO‐8711 (EP1), PF‐04418948 (EP2), L‐798106 (EP3), and L‐161982 (EP4)), the same procedure was utilized with vincristine ranging from 0 to 50 nm and indomethacin or EP antagonists ranging from 0 to 20 μm (the data were analyzed with synergyfinder 3.0 [19]). To calculate the most synergistic area, the highest vincristine concentration (100 nm) was removed from the mPGES‐1 inhibitors and celecoxib.

### 2.6 Calcein AM efflux assay

To investigate the potential effect of the mPGES‐1 inhibitor 934 on the ABC transporters, incubation experiments with calcein AM were carried out. Briefly, SK‐N‐AS cells (20 000 cells/well) were seeded into black culture 96‐well 2D plates (CLS3603; Corning) and grown overnight to confluence. The next day, 50 μL of each compound (934, 118, celecoxib, indomethacin (at 0.04–40 μm), and verapamil (at 0.07–70 μm)) were diluted in cell culture medium, added to monolayers, and incubated at 37 °C for 1 h. Calcein AM (14948; Cayman) in 100 μL PBS was added to each well at a final concentration of 0.3 μm and the plate was further incubated for 1 h. Subsequently, cells were washed three times with 100 μL ice‐cold FBS‐free medium. The intracellular calcein fluorescence (excitation = 485 nm, emission = 535 nm) was measured in PBS (100 μL) with a SpectraMax® iD5 Multi‐Mode Microplate Reader (Molecular Devices, San Jose, CA, USA). The signal from each well was normalized to the average signal of the DMSO‐treated control on the same plate. The intracellular diffusion and the subsequent release of calcein were monitored using the IncuCyte® S3 System. To determine the level of retention of calcein upon different treatments, cells were preincubated with compounds and calcein AM as described above. After media were replaced, cells were monitored in the Incucyte for 20 h. Area under the curve for each treatment and experiment was normalized to the positive control verapamil in respective experiment.

### 2.7 Immunohistochemical analysis of MRP1 and MDR1 in SK‐N‐AS/NHDF spheroids

SK‐N‐AS and NHDF cells were seeded in ULA 96‐well plate to form spheroids for 4 days. Spheroids were then harvested (collected in a tube using gravitational force only to remove supernatants in the following steps), washed in PBS, and fixed in 4% PFA for 1 h, followed by submersion in 70% ethanol and storage at 4 °C until paraffin embedding and sectioning. Sections were submerged in 3 × 5 min xylene to remove paraffin, followed by 5 min each in 99.5%, 95%, and 70% ethanol. After subsequent short washes in tap water and deionized water, antigen retrieval was performed using a pressure cooker and Tris‐EDTA, pH 9 buffer (10 mm Tris base, 1 mm EDTA, and 0.05% Tween 20). Slides were cooled down for 1 h, washed in PBS, and incubated 10 min in PBS‐SAP (0.1% Saponin (Thermo Scientific, Waltham, MA, USA), pH 7.4). To block endogenous peroxides, slides were incubated in 1% H_2_O_2_ in PBS‐SAP for 1 h, followed by 3 × 3 min washing in PBS‐SAP (if not otherwise stated, this will be the washing procedure between all the following steps). Biotin was blocked using an Avidin/Biotin blocking kit (SP‐2001, Vector, Stuttgart, Germany, 15 min incubation with each reagent with washing in between). Multidrug resistance‐associated protein 1 (MRP1) antibody (ab260038, 0.6 mg·mL^−1^; abcam, Cambridge, UK) was diluted 1 : 1000 (multidrug resistance protein 1 (MDR1), ab170904; abcam was diluted 1 : 1200) in PBS‐SAP supplemented with 3% normal human serum and incubated overnight at 4 °C. One section was also incubated with rabbit IgG at the same concentration. Following washing, sections were blocked with 3% goat serum for 15 min, immediately followed by biotinylated second antibody (goat anti‐rabbit, 30 min, BA‐1000, Vector) diluted 1 : 1600 in PBS‐SAP supplemented with 3% normal human serum and 1% goat serum. Slides were incubated with ABC elite kit (PK‐6100, Vector) for 45 min prior to development with DAB substrate kit (SK‐4100, Vector, 6 min), washed in PBS, and counterstained with Mayer's hematoxylin.

### 2.8 Gene expression analysis of ABCC1 and ABCB1 in SK‐N‐AS cells

A total of 200 000 SK‐N‐AS cells were seeded in six‐well plates, allowed to attach for 24 h, and subsequently treated with vincristine as single treatment or in combination with 934 for 24 h.

Total RNA from SK‐N‐AS cells was extracted using RNeasy Plus Micro Kit (Qiagen, Hilden, Germany; 74034) according to the manufacturer's instructions. After quantification with Nanodrop 2000 (Thermo Scientific), 500 ng total RNA was subjected to reverse transcription using a SuperScript™ VILO™ cDNA Synthesis Kit (ThermoFisher; 11754050) on a Veriti Fast 96‐W Thermocycle PCR machine (Thermo Fisher). Real‐time quantitative PCR (RT‐qPCR) was performed using Taqman probes (ABCB1, Cat#: 4453320 Assay ID: Hs00184500_m1; ABCC1, Cat#: 4453320 Assay ID: Hs01561483_m1; RPL13A, and Cat# 4448485 Assay ID: Hs03043885_g1) with either TaqMan™ Gene Expression Master Mix (ThermoFisher; 4369016) or a TaqMan™ Fast Advanced Master Mix (ThermoFisher; 4444556) in a CFX384 real‐time PCR detection system (Bio‐Rad, Hercules, CA, USA). Relative mRNA levels were analyzed using ∆∆*C*_t_ methods [20] by normalization to housekeeping gene RPL13A. Results are presented as fold change compared to solvent control (0.1% DMSO).

### 2.9 Gene expression analysis

Gene expression analysis of *ABCC1*‐encoding MRP1 was made using the R2: Genomics Analysis and Visualization Platform. Two public datasets were used: the Versteeg‐88 dataset with mRNA from 88 NB patients and the Kocak‐649 dataset with mRNA from 649 NB patients with survival data from 476 patients. The Kaplan Scan function was used to generate Kaplan–Meier curves with optimal survival cut‐off.

### 2.10 Statistical analysis

One‐way ANOVA with Tukey's or Dunnett's multiple comparisons test was used as statistical test when comparing more than two groups. Multiple comparisons were only analyzed if *P* < 0.05. Unpaired *t*‐test was used to compare two groups. AUC was used to compare retention of calcein over time. For statistical significance, *P < 0.05, **P < 0.01, ***P < 0.001, and ****P < 0.0001.

## 3 Results

### 3.1 Drug screening

**Fig. 1:**
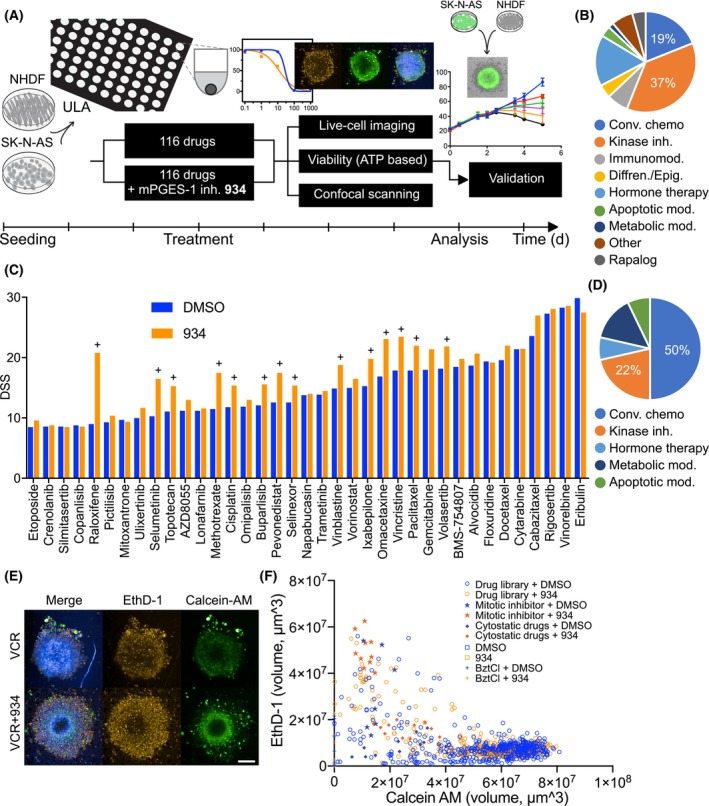
Combination screening of mPGES‐1 inhibitor and drugs using MCTS reveals effective combinations. (A) SK‐N‐AS cells and NHDF were seeded and MCTS formed. A library of 116 anticancer drugs was added at five concentrations as a single treatment or in combination with mPGES‐1 inhibitor 934. Plates were monitored during treatment using live cell imaging. After 4 days of treatment, dyes (Hoechst, a live cell dye (Calcein AM) and a cell death probe (Ethidium Homodimer I (EthD‐I)) were added and incubated for 3 h. The MCTS were then analyzed in a high‐content confocal imager. Finally, viability was assessed using an ATP‐based assay. (B) Drug library composition. Conv, conventional; chemo, chemotherapeutic; inh, inhibitors; mod, modulators; differen, differentiation; epig, epigenetic. (C) DSS > 8 was used as a cutoff for a single effect. To achieve a combination effect, the addition of the mPGES‐1 inhibitor 934 should increase the DSS score by more than 20% (marked as + in the graph), *n* = 1. (D) Annotation of combination hits based on the ATP‐based viability assay. Compared to the original drug library composition, more conventional chemotherapeutic agents were found among the combination hits. (E) Confocal images (maximum projection) of MCTS treated with 10 nm vincristine (VCR) or with VCR (10 nm) +934 (1 μm) and labeled with Calcein AM, EthD‐1, and Hoechst, *n* = 3. Scalebar = 200 μm. (F) Summary of image analysis. Each data point corresponds to a spheroid treated with drug at one concentration + vehicle control (drug library + DMSO, blue open circles) or drug + mPGES‐1 inhibitor 934 (drug library + 934, orange open circles). Spheroids treated with the positive control (BztCl) are indicated with a + sign, spheroids treated with the negative control (DMSO) are indicated with blue squares, and spheroids treated with 934 alone are indicated with orange squares. Some mitotic inhibitors are labeled with a star (including vincristine, ixabepilone, paclitaxel, vinblastine, vinflunine, docetaxel, and cabazitaxel), and some cytostatic drugs (including selumetinib, doxorubicin, fludarabine, gemcitabine, cytarabine, floxuridine, and methotrexate) with a diamond.

In a previous study, we showed that mPGES‐1 inhibitor, 934, enhanced the effect of vincristine in an MCTS model [14]. In the present study, we evaluated the compatibility of our model with drug screening using high‐content analysis (Fig. 1A). We also investigated whether inhibition of mPGES‐1 would enhance the effects of other drugs. Screening was performed using a drug library consisting of 116 drugs, ranging from approved cancer therapeutic drugs to experimental compounds belonging to several drug classes, such as conventional chemotherapeutic drugs, kinase inhibitors, metabolic modulators, and apoptotic modulators (Fig. 1B and Table S1). Each drug in the library was present in five concentrations covering a 10 000‐fold concentration range. First, conditions were established to downscale our established MCTS modeled to 384‐well plates. Then, co‐MCTS were treated with the drug library in the presence of 934 or vehicle control for 4 days. The MCTS were labeled with a mixture of probes measuring cell death and metabolic activity before imaging in a high‐content confocal screening microscope. Finally, ATP content was measured as a surrogate for viability, and the ATP‐based drug response was assessed by calculating a DSS. The screening revealed that 37 of 116 agents showed an effect as a single‐agent treatment (i.e., DSS > 8). The agents with the highest single‐agent DSS were cytarabine, cabazitaxel, rigosertib, vinorelbine, and eribulin (DSS of 21–30, Fig. 1C).

For a combination effect with 934, a 20% increased DSS was set as the cutoff value. Of the 37 drugs with a single effect (DSS > 8), 14 drugs met this condition and showed a potentiating effect with 934 compared with the vehicle control. Half of the drugs that showed a combination effect were among the conventional chemotherapeutic agents, although they accounted for only 19% of the drug library tested. In contrast, kinase inhibitors accounted for 37% of the drug library tested, but only 22% of the combination hits. Interestingly, among the hits that belonged to conventional chemotherapeutic agents, four of seven were mitotic inhibitors (Fig. 1D). Vincristine, which was evaluated for combination effects in our previous study, showed a 31% increased DSS when combined with 934 (i.e., the IC50 value for vincristine as single treatment was 21 nm compared with 7 nm for the combination of vincristine + 934).

High‐content confocal scanning was used to assess metabolic activity and cell death in spheroids after treatment, which provided additional information to the ATP‐based viability assay (Fig. 1E,F). Calcein AM was used to assess viable (metabolically active) cells and to measure the size of the spheroid as the volume of the fluorescent signal. The total volume of EthD‐1 fluorescent signal was also measured in the wells to assess cell death. The volume of calcein AM for each spheroid was correlated with the volume of EthD‐1 fluorescent signal (Fig. 1F). Spheroids were filtered out of the analysis if either calcein AM or EthD‐1 data were missing. A total of 1100 of the 1230 scanned spheroids were included in the image analysis. Drugs known for their cytotoxic effects on cancer cells, such as mitotic inhibitors, showed low calcein AM and high EthD‐1 signal, as expected. Drugs known for their cytostatic properties, such as selumetinib and gemcitabine, showed low calcein AM and low EthD‐1 signal, and DMSO controls showed high calcein AM and low EthD‐1 signal. One observation we made during image analysis was that the increase in EthD‐1 signal was higher for mitotic inhibitors than with cytostatic drugs in library + 934‐treated spheroids compared with library + DMSO‐treated spheroids. This confirms the results of the ATP‐based viability assay, in which mitotic inhibitors were enriched among the combination hits. We also compared the results of the viability assay with the results of image analysis by recording luminescence with calcein AM or EthD‐1. For the first 58 drugs, including most classical chemotherapeutic agents and most mitotic inhibitors, there is a positive correlation between calcein AM and the ATP‐based assay and a negative correlation between EthD‐1 and ATP production. Of note, for some mitotic inhibitors, viability decreases (and EthD‐1 signal increases) when treated in combination with mPGES‐1 inhibitor 934, while at the same time calcein AM signal increases (Fig. S2A,B).

When we established the MCTS system in our previous study, we examined the localization of fibroblasts and found that they were actively recruited to the center of the spheroid [14]. In the present study, we used more refined methods revealing that fibroblasts were not only localized in the center of the spheroid as previously thought but also populated the outermost layer explaining the superior structure of SK‐N‐AS/NHDF co‐MCTS compared with SK‐N‐AS mono‐MCTS (Fig. S3A). The presence of fibroblasts on the surface of the spheroid probably confers additional stability to the spheroid and thus explains the better structure of NB MCTS, which was found when co‐cultured with fibroblasts. We also treated the SK‐N‐AS/labeled NHDF spheroids to investigate whether the composition or localization of fibroblasts changed with treatment. Confocal images show that the fibroblast content of the spheroids did not change with treatment, but rather the unlabeled component, the tumor cells, was reduced. This supports our hypothesis that only the tumor cells are killed by treatment, although 934 mainly targets mPGES‐1 in the fibroblasts (Fig. S3B) [14].

### 3.2 Validation of hits

**Fig. 2:**
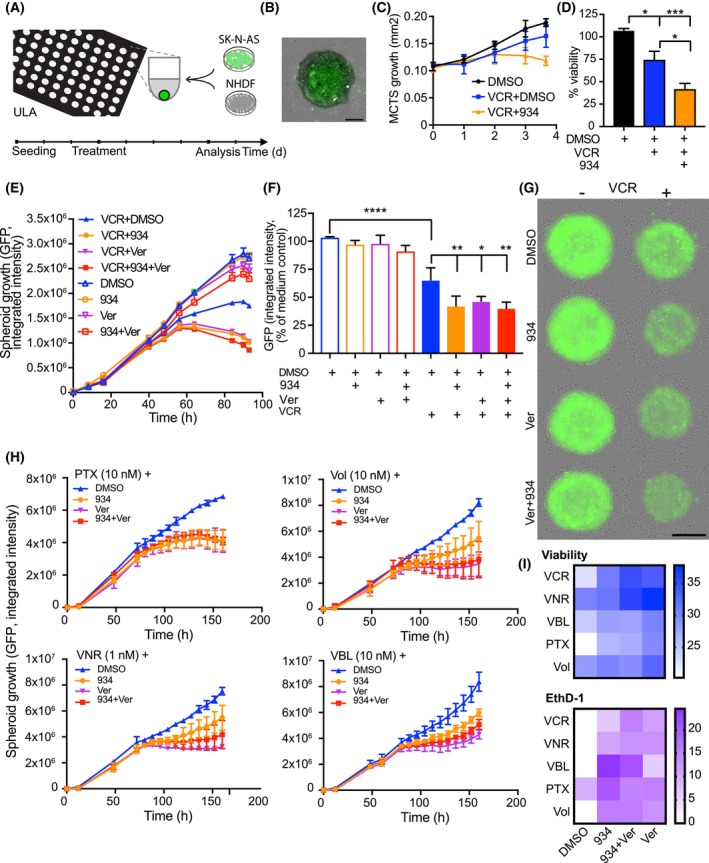
Validation method development and validation of combination drug screening. (A) Schematic overview of the experiment. SK‐N‐AS cells transfected with GFP were seeded together with NHDF, incubated for 3 days, and then treated with drugs or drug combinations and analyzed for another 4 days using live‐cell imaging and a confocal scanner. (B) Representative image, green fluorescence, and bright‐field overlay of GFP‐labeled MCTS acquired with a confocal scanner. Scale bar = 200 μm. The area of green fluorescence was measured and quantified over time using the IncuCyte® S3 system. The growth curve shows the mean cross‐sectional area ± SD of three spheroids in a representative experiment (C), and viability (D) was determined using CellTiterGlo 3D. ATP production was normalized to the untreated MCTS in four (*n* = 4) independent experiments with 3–8 spheroids/condition and experiment, mean ± SEM. One‐way ANOVA with Tukey's multiple comparisons test was used to calculate statistical significance. *P* (DMSO vs. VCR) = 0.02, *P* (VCR vs. VCR + 934) = 0.02, and *P* (DMSO vs. VCR + 934) = 0.0002. *P < 0.05, ***P < 0.001. (E–G) MCTS consisting of GFP‐labeled SK‐N‐AS cells and unlabeled NHDF seeded in 96‐well spheroid plates were treated for 3 days with vehicle (DMSO), 934 (2 μm), verapamil (Ver, 10 μm), VCR (1 nm), 934 + Ver, VCR + 934, VCR + Ver, or all three drugs together (VCR + 934 + Ver). Three independent experiments were performed. MCTS were monitored in the IncuCyte®S3 system, and green fluorescence and bright‐field images were acquired. The growth curve (E) shows a representative experiment (mean total integrated volume intensity ± SD of 4–8 MCTS). In (F), the endpoint size of spheroid is shown as integrated intensity of green fluorescence normalized to untreated control MCTS, combining all three experiments (mean ± SD, *n* = 3, 4–8 MCTS/experiment and group, *P* (DMSO vs. VCR) < 0.0001, *P* (VCR vs. VCR + 934/ver/934 + ver) = 0.009/0.04/0.004, respectively, one‐way ANOVA with Tukey's multiple comparisons test). *P < 0.05, **P < 0.01, ****P < 0.0001. (G) Phase contrast/green fluorescence overlay of representative spheroids from one experiment showing decreased area and intensity of green fluorescence in spheroids treated with the combination of VCR and 934/Ver/Ver + 934 compared to VCR alone. Scale bar = 200 μm. (H) Fourteen drugs, twelve with combination effect and two with single effect, were selected from the drug screen based on ATP production. GFP‐labeled SK‐N‐AS cells and unlabeled NHDF were seeded in 384‐well spheroid plates and 3 days after seeding MCTS were treated with each drug as a single treatment (+vehicle), in combination with 934 (2 μm), Ver (10 μm), or both 934 + Ver. Treated MCTS were observed for 4 days in the IncuCyte® S3 system, with both bright‐field images and green fluorescence images acquired. Growth curves of selected concentrations of paclitaxel (PTX), volasertib (Vol), vinorelbine (VNR), and vinblastine (VBL) are shown as mean integrated total green fluorescence signal ± SD of two MCTS in one experiment. (I) The experiment in (H) was repeated for VCR, VNR, VBL, PTX, and Vol with unlabeled SK‐N‐AS/NHDF spheroids (three spheroids/group). DSS was calculated from both viability (CellTiterGlo assay) and cell death (EthD‐1 imaging) for the different combinations and shown in a heatmap.

To validate the screening results, our strategy was to first reduce the number of data points by scoring them by ATP production and then perform a counter‐screening using GFP‐labeled SK‐N‐AS cells/NHDF MCTS (MCTS^GFP^). The MCTS^GFP^ system (Fig. 2A,B) allowed us not only to perform a kinetic assay but also to study the effect of the drugs on the cancer cells themselves. After GFP‐labeled SK‐N‐AS cells were generated, the effect of treatments on the MCTS^GFP^ system was evaluated. Simultaneous treatment of MCTS^GFP^ with vincristine and 934 resulted in enhanced cytotoxic effects, as compared to single agent treatments (i.e., decreased viability and decreased GFP signal over time, Fig. 2C,D). Calcein AM, used to measure metabolic activity in the drug screen, is a substrate of certain multidrug resistance proteins, and when we analyzed the confocal images from the initial screening, we found that the calcein AM signal increased with some drugs, for example, vincristine and paclitaxel in combination with 934, while still being associated with decreased viability and increased cell death. We, therefore, investigated the possibility that the mPGES‐1 inhibitor 934 might affect intracellular drug concentration by blocking ABC transporters, for example, MRP1 or MDR1. Spheroids were treated with vincristine in combination with 934 and/or verapamil. Verapamil is a calcium channel blocker acting as a classical chemosensitizer and substrate of MDR1, which is known to enhance the antitumor effect of therapeutic agents in various cancer cells, including NB. The effect of the drugs was monitored and quantified over time in the IncuCyte® S3 system using the green fluorescence signal of the MCTS^GFP^. The combination of vincristine + verapamil or vincristine + verapamil + 934 had the same effect as vincristine + 934 on spheroid growth (Fig. 2E–G). The images also show the loss of GFP signal in dead cells surrounding the live spheroid core (Fig. 2G).

After pre‐selection based on ATP production, 12 of the hits (buparlisib, cisplatin, paclitaxel, selinexor, vinblastine, pevonedistat, methotrexate, omacetaxine, volasertib, selumetinib, raloxifene, and topotecan) were selected, as well as two drugs with high single effect but no combination effect (rigosertib and vinorelbine). Since we had observed comparable treatment results with MCTS^GFP^ as for unlabeled MCTS in our previous experiments, we used MCTS^GFP^ to validate the combination hits. The selected drugs were also tested in the presence of verapamil. The combination effect of 934 was found to potentiate the efficacy of four validated drugs, namely vincristine, paclitaxel, volasertib, and vinblastine (Fig. 2H and 2E).

For paclitaxel (Fig. 2H), as with vincristine (Fig. 2E), the combination with verapamil or verapamil + 934 resulted in the same GFP intensity. Vinorelbine, which had a high single effect on ATP production in the initial screen, but no additional combination effect, showed an enhanced effect when combined with 934 in the GFP validation experiment (Fig. 2H). The high effect of vinorelbine alone on the screen, resulting in a small therapeutic window for any combination effects, could have masked any additional effect of the mPGES‐1 inhibitor. Addition of verapamil resulted in a slightly higher combination effect with vinorelbine, volasertib, and vinblastine when compared with the same drugs in combination with 934 (Fig. 2H). This suggests that time‐lapse imaging of drug treatment with GFP‐labeled SK‐N‐AS may reveal more nuanced differences between single‐agent treatments and combinations. In a further validation step, the combination treatments of vincristine, paclitaxel, vinblastine, vinorelbine, and volasertib with verapamil and 934 were repeated with unlabeled MCTS using the same imaging setup as in the screening experiment. DSS values were calculated based on viability (ATP production) and cell death (EthD‐1) (Fig. 2I). Viability results confirmed the MCTS^GFP^ experiment, with vincristine and paclitaxel showing the strongest combination effect and similar combination effects for all 934, verapamil, and 934 + verapamil. Vinorelbine showed the lowest combination effects with 934 and the highest combination effects with verapamil and verapamil + 934. Vinblastine showed the highest combination effect with verapamil, as in the MCTS^GFP^ validation. EthD‐1 imaging results showed good combination effects with all compounds.

The image analysis data from the initial screen show that the compounds whose combination effect with compound 934 was confirmed in the validation showed a greater increase in EthD‐1 than some of the compounds whose effect we could not validate in this system, that is, cisplatin, buparlisib, methotrexate, omacetaxine, selumetinib, pevonedistat, and selinexor (Fig. S2C). The greater sensitivity of the EthD‐1 compared with calcein measurement can in part be explained by the increased calcein signal in the presence of 934 which causes an overestimation of live cells in the imaging analysis. The remaining two screening hits, raloxifene and topotecan, could not be validated with MCTS^GFP^ or explained by image analysis data.

### 3.3 Synergistic effects of specific hits

**Fig. 3:**
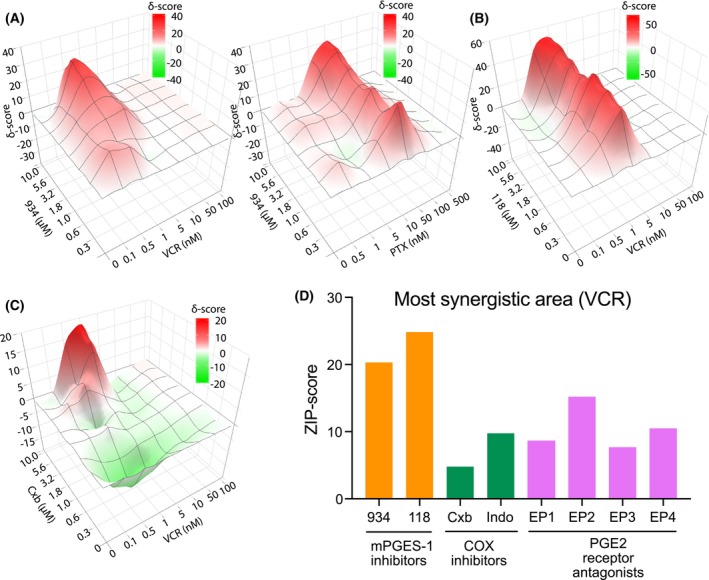
Comparison of synergy with inhibitors of enzymes/receptors in the COX/mPGES‐1/EP pathway. Two compounds, vincristine (VCR) and paclitaxel (PTX), were tested for synergy with mPGES‐1 inhibitor 934 (A). Another mPGES‐1 inhibitor, 118 (B), and a COX‐2 selective inhibitor, celecoxib (Cxb) (C), were also tested for synergy with VCR. ATP‐based viability was measured and analyzed using synergyfinder 2.0. Synergy with VCR was also assessed with the COX inhibitor indomethacin (Indo) and the PGE_2_ receptors EP1‐EP4 (Fig. S4). (D) The most synergistic area was calculated using synergyfinder 3.0. All synergy tests were performed once (*n* = 1) and individual concentrations were retested in at least three individual experiments.

Two of the validated drugs, vincristine and paclitaxel were selected for in‐depth synergy analysis with 934 (Fig. 3A). Co‐MCTS were, therefore, treated with eight concentrations of vincristine or paclitaxel in the presence (or absence) of eight concentrations of 934. ATP‐based viability was used as an indicator of treatment, and data were analyzed using Synergy Finder software and presented as ZIP score, which represents the deviation of data across the dose–response matrix compared with the expected outcome. The combination of 934 with vincristine resulted in an overall ZIP score of 9.4 with a maximum local ZIP synergy score of 20.5 (Fig. 3A,D). The combination of paclitaxel with 934 resulted in an overall ZIP score of 8.1 and a maximum synergy score of 17.9 (Fig. 3A).

To verify whether the synergy was specific to 934 or related to the effect of mPGES‐1 inhibition, we tested vincristine in combination with another mPGES‐1 inhibitor 118, a structurally related compound [15]. Interestingly, vincristine in combination with 118 resulted in a synergistic ZIP score of 12.8 with a local maximum of 25.1 (Fig. 3B,D). In addition, we used celecoxib, a COX‐2 selective drug, and indomethacin, a non‐selective COX inhibitor that inhibits all prostaglandins including PGE_2_, together with vincristine as reference drugs. To our surprise, the total ZIP score for celecoxib and vincristine was only 0.3, with an observed maximal local synergy score of 5.0 and 2.9 for indomethacin and vincristine with the most synergistic area of 10.0 (Fig. 3C,D and Fig. S4B). Because COX inhibition did not result in the same level of synergy as mPGES‐1 inhibition, we decided to test PGE_2_ receptor antagonists in combination with vincristine. Of the four antagonists tested, the EP2 antagonist PF‐04418948 showed the highest local ZIP value (Fig. 3D and Fig. S4A). To verify the synergy test, spheroids were treated with 1 nm vincristine in combination with a fixed concentration of celecoxib, indomethacin, or EP antagonists. 934 was used as a positive control. Only 934 in combination with vincristine significantly decreased spheroid viability compared with vincristine treatment alone confirming the lower synergistic results obtained with COX inhibitors and EP antagonists (Fig. S4C).

### 3.4 Effect of mPGES‐1 inhibitors on multidrug resistance proteins

Given the high local synergy of mPGES‐1 inhibitors 934 and 118 and the low synergy of COX inhibitors with vincristine, and the general potentiation of 934 on vincristine and paclitaxel observed in our MCTS model, we decided to investigate the involved underlying biological mechanism. Binding of 934 to tubulin and thus affecting mitosis was excluded as a possible mechanism of action, as no stabilization of β‐tubulin was observed (CETSA experiments using fixed temperature [21], data not shown).

**Fig. 4:**
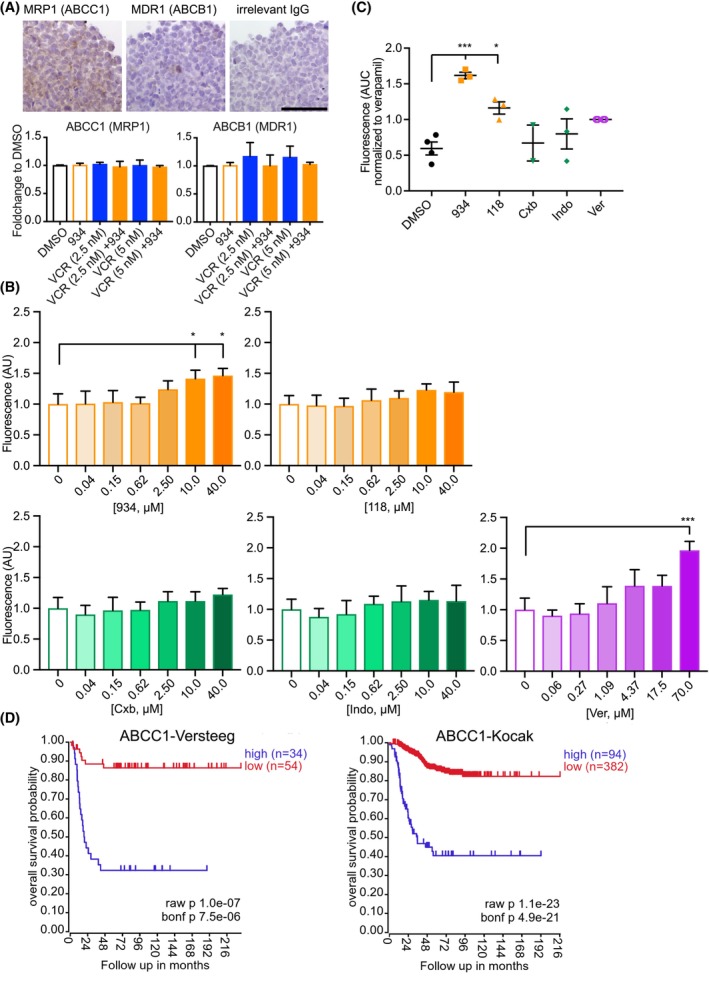
Multidrug resistance proteins. (A) Expression of MRP1, MDR1 (immunohistochemical staining, Scale bar = 100 μm), ABCC1 (MRP1), and ABCB1 (MDR1) mRNA. Mean ± SD of three independent experiments with two to four technical replicates in each experiment. (B, C) Uptake and retention of fluorescent calcein by SK‐N‐AS cells in the presence of increasing concentrations of 934, 118, celecoxib (Cxb), indomethacin (Indo), or verapamil (Ver). Cells were treated with each drug for 1 h and then with calcein AM (0.3 μm) for an additional hour at 37 °C. The extracellular calcein AM was then washed off, and intracellular fluorescent calcein was measured with a plate reader (B). Each data point represents the mean ± SD of three independent experiments. *P* value denotes statistically significant difference compared with control (untreated cells). *P* (ver, 70 μm) = 0.0002, *P* (934, 10 μm) = 0.02, and *P* (p34, 40 μm) = 0.01. One‐way ANOVA, Dunnett's multiple comparisons test. *P < 0.05, ***P < 0.001. (C) Accumulation and subsequent retention of calcein were also measured by time‐lapse microscopy over 20 h, shown as area under the curve (AUC) normalized to positive control verapamil, in two to four independent experiments (*n* = 2 for Cxb, *n* = 3 for 934, 118, and Indo, and *n* = 4 for vehicle control and Ver; see Fig. S5 for results of individual experiments). All compounds were used at a final concentration of 10 μm, except verapamil, which was administered at a concentration of 70 μm. *P* (DMSO vs. 934) = 0.0002, *P* (DMSO vs. 118) = 0.03. (D) Analysis of *ABCC1* mRNA in two expression cohorts, Versteeg (*n* = 88) and Kocak (*n* = 476), shows that high expression of *ABCC1*‐encoding MRP1 is associated with worse overall survival in NB patients. Kaplan–Mayer plots were generated using R2: Genomics Analysis and Visualization Platform.

Considering our previous observation that the calcein AM intensity was increased when vincristine was combined with 934, that 934 and verapamil showed similar trends, and that verapamil is known to enhance intracellular retention of chemotherapeutic agents, effects on ABC transporters were then examined. Prior to measuring the effects on efflux pumps, we examined the expression of MRP1 (ABCC1) and MDR1 (ABCB1) in SK‐N‐AS/NHDF spheroids and monolayer SK‐N‐AS cells. Gene expression of both ABCC1 and ABCB1 was found in SK‐N‐AS cells. IHC analysis confirmed expression of MRP1 in the tumor cells also when grown as spheroids although only low expression of MDR1 could be detected (Fig. 4A). After protein and/or mRNA expression of ABCC1 and ABCB1 was established, the effects on pumps were examined by measuring ABCB1/ABCC1‐mediated calcein AM efflux in 2D‐cultured SK‐N‐AS cells to isolate the effect on tumor cells only. Because of the rapid calcein efflux combined with the slow penetration into MCTS, this was a more feasible approach. Calcein AM, a cell‐permeable, non‐fluorescent compound, is a known efflux transporter substrate that has been used to evaluate inhibitors or competitive substrates. In cells, calcein AM is metabolized to fluorescent calcein with low membrane permeability. Although calcein has a lower affinity for the ABC transporters compared to calcein AM, both compounds can be cleared from cells via these multidrug resistance pumps. Calcein in cells treated with 70 μm verapamil was significantly increased compared with cells treated with vehicle (DMSO), consistent with the activity of verapamil as an inhibitor of the ABC transporters. 934 (at 10 and 40 μm) increased the intracellular fluorescence (i.e., dye accumulation) compared with vehicle control (Fig. 4B). None of the other drugs (118, celecoxib, or indomethacin) had any effect at the concentrations tested (Fig. 4B). Interestingly when the net accumulation of the dye over time was considered, greater retention was observed with 934 and 118 (Fig. 4C and Fig. S5) than with the COX inhibitors and the vehicle control. In addition, gene expression analysis of *ABCC1*‐encoding MRP1 was made using the R2: Genomics Analysis and Visualization Platform. As shown in Fig. 4D, patients with tumors expressing high levels of *ABCC1* had lower overall survival compared to patients with low *ABCC1* expression levels. Taken together, these results suggest that the synergistic effect of 934 + vincristine, as well as 934 + paclitaxel, is mediated, at least in part, by inhibition of efflux transporters, which may have a clinical significance because high expression of *ABCC1* is associated with worse clinical outcome in NB patients.

### 3.5 Validation of combination hits using NB spheroids covering various phenotypic traits

**Fig. 5:**
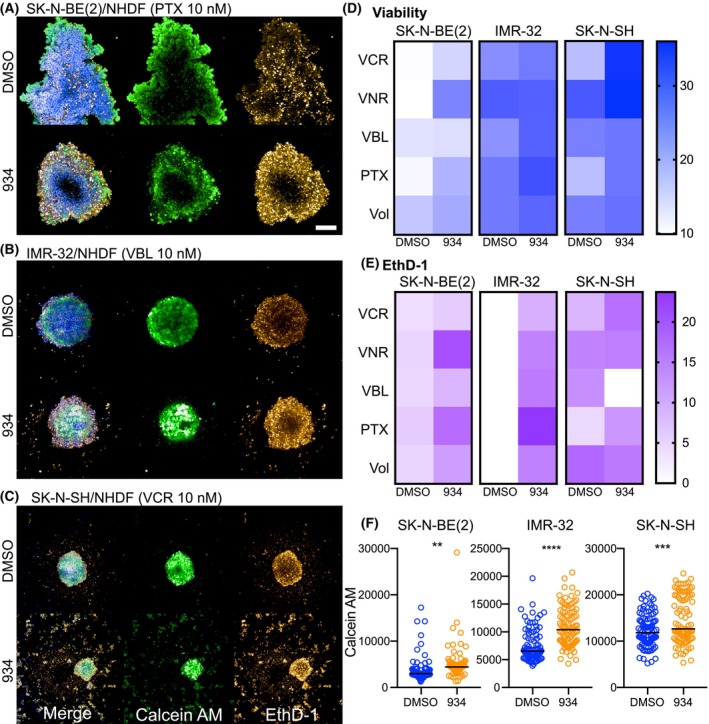
Validation with other NB cell lines. Spheroids were formed with NHDF and other NB cell lines, including IMR‐32, SK‐N‐BE(2), and SK‐N‐SH, treated with vincristine (VCR), vinorelbine (VNR), vinblastine (VBL), paclitaxel (PTX), and volasertib (Vol) at five concentrations (three spheroids/condition) as a single treatment (+DMSO) or in combination with 934 (2 μm) in one experiment. Confocal images show maximum projections of representative co‐MCTS of SK‐N‐BE(2) treated with 10 nm PTX (A), IMR‐32 treated with 10 nm VBL (B), or SK‐N‐SH, treated with 10 nm VCR (C), with 934 (2 μm) or vehicle control (DMSO). Spheroids were labeled with calcein AM, EthD‐1, and Hoechst. Scalebar = 200 μm. (D) Cell viability was measured using CelltiterGlo3D, and DSS was calculated and shown in a heatmap. (E) EthD‐1 signal for all concentrations was used to calculate DSS scores for the different combinations and visualized in a heatmap. (F) Mean calcein fluorescence signal in all individual spheroids for each cell line. Unpaired *t*‐tests were used for statistical analysis, *P* (SK‐N‐BE(2)) = 0.003, *P* (IMR‐32) < 0.0001, and *P* (SK‐N‐SH) = 0.0005. **P < 0.01, ***P < 0.001, and ****P < 0.0001.

To account for the heterogeneity of NB tumors and to test whether our main combination hits are effective in other NB cell lines, co‐MCTS were formed with NHDF and SK‐N‐BE(2), IMR‐32, and SK‐N‐SH, respectively. Both SK‐N‐BE(2) and IMR‐32 exhibit *MYCN* amplification and an adrenergic phenotype, whereas SK‐N‐SH cells with a mesenchymal phenotype show neither *MYCN* amplification nor 11q deletion, as do SK‐N‐AS (11q deletion, mixed but mostly adrenergic phenotype). Spheroids were treated with vincristine, paclitaxel, volasertib, vinblastine, and vinorelbine with or without mPGES‐1 inhibitor 934, using the same setup as described in Section 3.2 (Fig. 5). SK‐N‐BE(2)/NHDF spheroids were the most resistant to single treatments among the three co‐MCTS, and the most effective combinations with 934 were observed with vinorelbine, paclitaxel, and vincristine. Volasertib also proved to be a combination hit with a 20% increase in DSS when combined with 934, and the viability assay showed a high correlation with EthD‐1 imaging data. IMR‐32 co‐MCTS showed a high DSS in terms of viability for single‐agent treatment, with vinblastine and paclitaxel as combination hits with 934. In contrast to viability testing, low DSS values were obtained for EthD‐1 staining of single‐agent treated co‐MCTS. In the SK‐N‐SH co‐MCTS, vincristine and paclitaxel were identified as combination hits for both the viability assay and EthD‐1 staining. All three co‐MCTS showed an overall increase in calcein at 934 combination treatments compared with single treatment (Fig. 5F).

## 4 Discussion

Cancer is one of the leading causes of death worldwide and was responsible for nearly 10 million deaths in 2020 (World Health Organization (2 February 2022)). Despite tremendous progress in the development of cancer therapies for solid tumors, high failure rates are reported (~ 90%) and the main cause is attributed to reductionist research strategies [22]. The key element in new drug development starts with basic research and requires the appropriate use of preclinical *in vitro* models. 3D models consisting of cancer and stromal cells in a heterotypic environment can better replicate the human disease state and provide more meaningful data on how the therapy may work in clinical practice [23, 24, 25].

In our previous study, we cultured NB cells and NHDF in 3D tumor spheroids to mimic NB tumors in terms of COX/mPGES‐1/PGE_2_‐signaling pathway. We also demonstrated that selective inhibition of mPGES‐1 with inhibitor 934 reduced the production of PGE_2_ by NHDF *in vitro* [14]. *In vivo*, inhibition of mPGES‐1 suppressed the tumor‐promoting properties of the tumor microenvironment and resulted in reduced tumor growth *in vivo* [12].

In this study, we demonstrate that our MCTS model combined with imaging/cell viability testing is suitable for high‐throughput analysis of anticancer drug effects and testing of combination effects. To our knowledge, high‐content analysis of anticancer drug combinations has not been previously investigated using NB MCTS. Our screening allowed the identification of several clinical drugs from different therapeutic categories that have potentiating effects in combination with mPGES‐1 inhibitor 934. Interestingly, the active hits were mainly enriched with compounds from conventional chemotherapeutic categories, described in a recent publication to upregulate PGE_2_ production [26]. Several of the active hits were previously tested *in vivo* (e.g., in bladder, breast, and colon tumor models) in combination with EP4 receptor antagonists or celecoxib (COX‐2 inhibitor) [9, 10]. Similar to our results with 934, increased chemosensitivity of tumors to the combination treatments was reported [9, 10, 14]. This could be partially explained by the inhibition of PGE_2_‐mediated expansion of cancer stem cells. While chemotherapy triggers tumor cell death, the associated release of PGE_2_ paradoxically activates proliferation of existing dormant cancer stem cells, contributing to tumor recurrence [9, 27, 28]. Therefore, inhibition of PGE_2_ could improve tumor response to chemotherapy by reducing the number of cancer stem cells available to repopulate the tumor [10]. This has been studied in detail in urothelial carcinoma xenografts, where inhibition of PGE_2_ release reduced the paracrine effects of adjacent dying cells and blocked the reawakening of quiescent cancer stem cells [9]. Interestingly, celecoxib (a COX‐2 selective inhibitor) effectively prevented subsequent wound‐induced tumor repopulation and attenuated the progressive manifestation of chemoresistance in primary xenografts derived from a patient resistant to chemotherapy [9]. The authors demonstrated that urothelial carcinoma cells are the main source of PGE_2_ and that chemotherapy‐induced apoptosis of cancer cells is closely associated with its release. However, PGE_2_ has been shown to be produced not only by dying cancer cells but the sources of PGE_2_ are heterogeneous and depend on the type of tumor also [29]. For example, our previous studies have shown that CAFs are the major source of PGE_2_ in NB [11, 12]. Selective inhibition of mPGES‐1 reduced CAF‐derived PGE_2_ production in preclinical NB mouse models, thereby reducing tumor growth [12]. Regardless of the source, 934 most likely potentiates the effect of chemotherapeutic agents by blocking the mPGES‐1/PGE_2_ pathway. This was also confirmed by studying the synergy between vincristine or paclitaxel and another mPGES‐1 inhibitor (118, a compound structurally related to 934). Because all combinations of mPGES‐1 inhibitors (vincristine + 934, paclitaxel + 934, and vincristine + 118) showed a synergistic effect, inhibition of PGE_2_‐mediated repopulation of cancer cells could be considered an important element of the observed tumor chemosensitivity. To our surprise, the combination of vincristine + celecoxib, a COX‐2 selective inhibitor, did not show the same degree of synergy as the mPGES‐1 inhibitors at PGE_2_ inhibitory concentrations, and only moderate synergy was observed with vincristine + indomethacin, a non‐selective COX inhibitor. The EP2 antagonist PF‐04418948 exhibited the highest synergy among the EP antagonists tested, in the same range as 934, although it failed to reduce spheroid viability at five times the concentration of 934 when combined with 1 nm vincristine compared to 934. These results suggest that the combination effects may not be mediated by PGE_2_ alone but may also be due to a shunting effect to other prostaglandins or off‐target effects of 934 and its structurally related compound 118.

Another plausible mechanism is the blocking (off‐target effect of 934) and/or decreased expression (PGE_2_ mediated) of ABC transporters, for example, MDR1 (also called P‐glycoprotein or ABCB1) and MRP1 (ABCC1). These multidrug resistance proteins efflux chemotherapeutic agents from cancer cells, leading to major problems with chemoresistance and resulting in relapses [30, 31, 32]. It has been reported that high levels of ABC transporters contribute to drug resistance in various cancers (e.g., liver and breast cancer) and have a significant impact on clinical outcomes [10, 33]. Activation of COX‐2 increases the expression and activity of many ABC transporters and plays an important role in the elimination of drugs from cells [34, 35, 36, 37, 38]. For example, PGE_2_ has been shown to induce MDR1 expression, and celecoxib inhibits MDR1 expression through a COX‐2‐dependent mechanism in human hepatocellular carcinoma cell lines [33, 39]. Similar observations were made for EP4 antagonism, that is, a significant decrease in BCRP, MRP2, MRP3, and MRP4 expression [10]. To support the hypothesis that the mPGES‐1 inhibitor 934 attenuates chemotherapy‐induced ABC transporter‐dependent drug resistance, drugs potentiated in the screen by 934 were also tested in the presence of verapamil, a calcium channel blocker and classical chemosensitizer known to decrease chemoresistance in various cancer cells including NB [40, 41]. As expected, verapamil enhanced the chemotherapeutic effect of the drugs in a manner similar to 934.

Therefore, to further elucidate the effects of 934 on ABC transporters, active extrusion of calcein AM (MDR1 substrate) and intracellular accumulation of calcein (an anionic dye and substrate for MRP1) in MCTS were investigated [34, 42, 43]. When comparing calcein intensity in some of the hits selected for validation, MCTS treated with 934 showed a higher fluorescence signal compared to MCTS treated with vehicle and the respective drug, although viability was reduced and, in some cases, even with increased EthD‐1. Therefore, we speculate that the chemo‐sensitizing potential of 934 in MCTS may be due in part to inhibition of both MRP1 and MDR1. To complement these experiments and to better understand the effect of the mPGES‐1 inhibitor 934 on the ABC transporters, the effect on calcein uptake/efflux was studied in SK‐N‐AS 2D cultures. Considering that vincristine and paclitaxel are known substrates of MRP1 and MDR1, respectively [34, 44, 45], we hypothesized that if 934 blocked these transporters, the intracellular concentration of the drugs would be higher and lead to increased cell death. Interestingly, 934 not only decreased efflux of calcein AM (blocked MDR1) but also increased accumulation of calcein (inhibited MRP1). Verapamil efficiently inhibited MDR1 but showed minimal effect on MRP1 compared to 934, consistent with its known published effects [46, 47]. Interestingly, none of the other COX/mPGES‐1/PGE_2_ inhibitors showed any effect on MDR1. This suggests that either their effects are not mediated by inhibition of this specific transporter or that their minimally effective concentrations are much higher than those of 934. However, only a small proportion of cells were positive for MDR1 at the protein level. Treatment with mPGES‐1 inhibitors 934 and 118, but not COX inhibitors, maintained the calcein fluorescence signal for a prolonged period, suggesting inhibition of MRP1. The superior ability of the mPGES‐1 inhibitors tested to block MRP1 may explain the difference between celecoxib and 934 observed in achieving synergistic effects together with vincristine, a known target of MRP1. Overall, 934 reversed drug resistance likely by inhibiting or downregulating the activities of ABC transporters (MRP1 and MDR1). MRP1 expression was confirmed in SK‐N‐AS cells in both monolayer culture (mRNA expression) and co‐MCTS (protein level). Although no change in mRNA levels was observed in the treated cells, this needs to be further investigated, for example, with more time points and concentrations, to draw conclusions.

Finally, the combination hits were tested on additional NB co‐MCTS with SK‐N‐BE(2), IMR‐32, and SK‐N‐SH cells to investigate the generality of the combinations from the SK‐N‐AS/NHDF co‐MCTS screen and to address heterogeneity in NB tumors. Imaging revealed differences both in the morphology of the spheroid and which of the combination hits were effective in corresponding co‐MCTS. Interestingly, calcein AM signal was increased in all three co‐MCTS when the drugs were combined with the mPGES‐1 inhibitor 934, suggesting that efflux pump blocking may be responsible for the observed combination effect, as in SK‐N‐AS co‐MCTS.

Overall, our high‐throughput drug screening platform enabled effective identification of single and combination responses in the presence of an mPGES‐1 inhibitor. Although synergistic drug combinations identified by different readouts (viability and GFP signal) showed high overall agreement in our initial screening, some positive hits from the ATP‐based readouts could not be validated with the GFP‐transfected MCTS. This highlights the need to evaluate multiple parameters when screening drug/combinations to validate results rather than relying on one assay alone. The temporal dimension provided by the use of GFP‐transfected MCTS and live‐cell imaging also contributed to the screening endpoint measurements with a more nuanced readout. In addition, we demonstrated that at least part of the synergistic effect of 934 and vincristine (or paclitaxel) was due to inhibition of the ABC transporters (MRP1 and MDR1). The synergistic drug combinations would not only increase the efficacy of the antitumor agent but could also decrease the dosage to reduce side effects. Although 934 is a probe compound and therefore not optimized for *in vivo* pharmacokinetics or pharmacodynamics, there are ongoing clinical investigations with other mPGES‐1 inhibitors outside of cancer. Evaluation of alternative inhibitors in future studies can help strengthen the mechanistic observations and the role of PGE_2_ in chemoresistance.

Although not all of the technical issues with using spheroids to screen drugs have been resolved, a paradigm shift is emerging in the way cancer drug discovery will be conducted in future studies. Better 3D *in vitro* cancer models will hopefully help increase the relatively low success rate of new cancer drugs (the estimated approval rate of drugs entering phase I in oncology is only 5.3%) [48].

## 5 Conclusion

In conclusion, the present work highlights the importance of screening drug combinations with MCTS and the need for careful selection of the most appropriate hits for further preclinical testing. To this end, we developed a cost‐effective model and tested different selection methods to increase the robustness of our results. Further analysis of the effects of mPGES‐1 inhibitors and chemotherapeutic agents is needed to better understand the mechanism of the synergies found.

## Conflict of interest

Per‐Johan Jakobsson is a co‐founder of Gesynta Pharma AB, a company that develops mPGES‐1 inhibitors. The other authors declare no conflict of interest related to the content of this study.

## Author contributions

KL and BS‐L conceived and designed the project; AZ, EA, LJ, MS, JS‐S, BS‐L, and KL acquired and interpreted the data; FW, PK, MK, PÖ, P‐JJ, BS‐L, and KL supervised and provided scientific input and resources; AZ, BS‐L, and KL wrote the original draft; and all authors read and approved the final manuscript.

### Peer review

The peer review history for this article is available at https://www.webofscience.com/api/gateway/wos/peer‐review/10.1002/1878‐0261.13502.

## Supporting information


**Fig. S1.** Comparison of different imaging parameters with the coefficient of variation.

**Fig. S2.** Comparison of image analysis parameters and viability assay.

**Fig. S3.** Organization of cells in MCTS.

**Fig. S4.** Additional comparison of synergy with inhibitors of enzymes/receptors in the COX/mPGES‐1/EP pathway.

**Fig. S5.** Calcein AM assay.

**Table S1.** Drug library.

## Data Availability

The data that support the findings of this study are available in figures, and/or the Supporting Information of this article. Detailed information about individual drugs and concentrations in Fig. 1F is available from the corresponding author (karin.larsson@ki.se) upon reasonable request.
